# Comparison of Cohesive Models in EDEM and LIGGGHTS for Simulating Powder Compaction

**DOI:** 10.3390/ma11112341

**Published:** 2018-11-21

**Authors:** Cristina Ramírez-Aragón, Joaquín Ordieres-Meré, Fernando Alba-Elías, Ana González-Marcos

**Affiliations:** 1 Department of Mechanical Engineering, University of La Rioja, C/Luis de Ulloa, 20, 26004 Logroño, Spain; maria-cristina.ramirez@alum.unirioja.es (C.R.-A.); fernando.alba@unirioja.es (F.A.-E.); ana.gonzalez@unirioja.es (A.G.-M.); 2 Department of Industrial Engineering, Business Administration and Statistics, Polythecnic University of Madrid, C/José Gutiérrez Abascal, 2, 28006 Madrid, Spain; 3 Visiting Scholar at École Polytechnique, Route de Saclay, 91128 Palaiseau, France

**Keywords:** powder compaction, discrete element method (DEM), cohesive contact models, LIGGGHTS, EDEM

## Abstract

The purpose of this work was to analyse the compaction of a cohesive material using different Discrete Element Method (DEM) simulators to determine the equivalent contact models and to identify how some simulation parameters affect the compaction results (maximum force and compact appearance) and computational costs. For this purpose, three cohesion contact models were tested: linear cohesion in EDEM, and simplified Johnson-Kendall-Roberts (SJKR) and modified SJKR (SJKR2) in LIGGGHTS. The influence of the particle size distribution (PSD) on the results was also investigated. Further assessments were performed on the effect of (1) selecting different timesteps, (2) using distinct conversion tolerances to export the three-dimensional models to standard triangle language (STL) files, and (3) moving the punch with different speeds. Consequently, we determined that a timestep equal to a 10% Rayleigh timestep, a conversion tolerance of 0.01 mm, and a punch speed of 0.1 m/s is adequate for simulating the compaction process using the materials and the contact models in this work. The results showed that the maximum force was influenced by the PSD due to the rearrangement of the particles. The PSD was also related to the computational cost because of the number of simulated particles and their sizes. Finally, an equivalence was found between the linear cohesion and SJKR2 contact models.

## 1. Introduction

Granular materials are compacted to form solid components in the powder metallurgy and ceramic industries. Some of the problems that occur during the compaction process are related to segregation of the materials, the weight variability or non-uniform density in compacts, and inadequate selection of the materials (without plasticity, lubricant, or binding agents) [1,2]. Using adequate simulation models may help engineers to address these constraints to develop new products.

The discrete element method (DEM) allows a variety of granular materials to be simulated by selecting an adequate contact model [3]. DEM has been used to analyse the mechanisms of segregation that occur when a hopper is filled or discharged with dry granules using non-cohesive contact models [4,5]. These contact models have also been used in other applications, such as to improve blending machines [6,7] and screeners [8,9]. These non-cohesive models may simulate elastic, elastic-plastic, or visco-elastic materials. The effect of the moisture content on particles is usually modelled by applying cohesive contact models that consider the Van der Waals forces to calculate the contact between the particles [10]. Other cohesive models are used to simulate the behavior of small-sized particles (powders) that tend to agglomerate by introducing an attraction force that pulls the particles toward each other, leading to the adhesion of the particles. Another contact model, called the bonded-particle model, establishes the bonds between the particles to keep them joined. Once the stress between two particles exceeds a critical value, the bond breaks. These models are used to simulate the mill process [11,12] or to determine the failure of structural materials [13,14].

Some of these contact models have been used to examine the behaviors of different materials during the formation of a compact. For example, Janda and Ooi [15] simulated the consolidation process of real soils by modelling a uniaxial confined compression. Then, they used the same model to analyse the penetration resistance of cohesive soils. Thakur et al. [16] simulated the compaction of detergent powders to form a cylindrical sample and analysed the relationship between the porosity of the compact and the consolidation stress. The results were contrasted with experimental data. Following this, an unconfined compression test was simulated. The stress-strain curves during failure were compared. Aranda [17] simulated the compaction of a refractory disc using different particle size distributions (PSDs) and materials. The porosity and permeability of the compacts were analysed in all cases. Other authors studied the effect of the PSD [18,19] and examined the influence of the particle shape [20,21] on the compaction process.

Different software packages for DEM simulations have been introduced. Some of them, such as EDEM^®^ (version 2018, DEM Solutions Ltd., Edinburgh, UK), ROCKY^©^ (version 4.1, ESSS, Florianópolis, Brazil), and PFC^TM^ (version 6.0, Itasca Consulting Group, Inc., Minneapolis, MN, USA), are commercially distributed, but other ones, like LIGGGHTS^®^ (version LIGGGHTS-PUBLIC 3.3.1, DCS Computing GmbH, Linz, Austria) or YADE (The Yade Project [22]), are open-source packages. All these software programs provide several contact models, so it is possible to choose the most appropriate program for simulating different processes or systems. For example, Jiménez-Herrera et al. [23] simulated the breakage of particle beds due to the impact of a ball using three contact models. Two of them were implemented in EDEM and the other in ROCKY. Some features, including the ease of using each model and the similitude of the simulation results with an experiment, were analysed. Another interesting approach is comparing the results using different simulation packages. Wei et al. [24] investigated the charging system of an ironmaking blast furnace using a non-cohesive model in EDEM and LIGGGHTS. The results obtained using each software program were compared. A good agreement was found between them and the experimental data. Similarly, Markauskas et al. [25] compared the results obtained using EDEM and DEMMAT (swMATH, Berlin, Germany) during a silo discharge. Soltanbeigi et al. [26] used the multi-sphere approach in EDEM and superquadric shapes in LIGGGHTS to simulate the behavior of non-spherical particles during different processes, such as heap formation, shear testing, or discharging of a silo. They also compared the results obtained using both software programs. Kozhar et al. [27] tested an irregular-shaped particle under uniaxial compression and then simulated the particle with DEM. They used two types of software for that purpose: EDEM and MUSEN (Institute of Applied Mechanics, Braunschweig, Germany). Two contact models were applied in EDEM, where a multi-sphere approach was used to simulate the irregular shape of the particle. In MUSEN, the particle was simulated with a bonded-particle model, where different models defined the characteristics of the bonds.

To satisfy the interest in the different DEM simulators and contact models, in this work, DEM was used to analyse the capability of some cohesion contact models to simulate the compaction of a granular mixture to form a compact. A system including a matrix and two punches was used for this purpose. Three cohesion contact models implemented in two software packages were applied to simulate the particles’ behavior. The results obtained using the different contact models were compared to determine their equivalence. The analysis considered the maximum force applied by the upper punch, the percentage of eliminated contacts after the ejection of the compact, and the computational costs required by the simulations. Three PSDs were modelled to observe the influence of the materials’ granulometric properties on the compaction process. The influence of some parameters, such as the timestep, geometries, and speed of the punch on the results, were studied. The optimal values of these parameters for simulating the compaction process using these contact models were determined. Consequently, these values could be used in future works to model the compaction of other materials.

Although the effects of the PSD or the speed of the punches on the compaction process are known, the novelty of this work is the use of different DEM simulators to compare several contact models and find equivalent models implemented in each DEM package. No references in the literature compare cohesive models implemented in different DEM simulators and apply them to the compaction of powders. Thereby, this study may be useful for those that use the commercial package EDEM, but also for people that use the open-source package LIGGGHTS. The results of this study provide a perspective on multiple simulation tools. This research might be interesting for future simulations of the compaction process because it provide some keys to defining the DEM models and reducing the computational costs of the simulations.

## 2. Materials and Methods

This section presents the characteristics of the DEM models that were simulated using EDEM and LIGGGHTS. It describes how the models were defined in each type of DEM software in order to prevent differences between both simulators, and presents the details about the methodology used in this work.

### 2.1. Discrete Element Method (DEM)

The discrete element method proposed by Cundall and Strack [28] is a numerical method that is capable of simulating the mechanical behavior of a granular medium that is composed of an assembly of particles that displace independently and interact with the other particles at their contact points. This method is based on the application of Newton’s second law of particles and a force-displacement law at the contacts. The properties of the particles (position, velocity, and forces acting on them) are updated at every timestep. At each timestep, a calculation cycle is completed. This cycle begins with the detection of contacts between particles. At this time, the contact point and the overlap between two particles in contact are calculated. Then, they are used to obtain the contact forces by applying the force-displacement laws. Once the forces and the moments applied to each particle have been calculated, the motion of each particle is calculated using Newton’s second law. Thereby, the acceleration, velocity, and positions of the particles are updated using a time integration algorithm, and the calculation cycle is completed. The updated values are used to detect the subsequent contacts and another cycle begins. Because an explicit integration technique performs the updates, the timestep must be very small to obtain stable numerical solutions. The maximum value of the timestep at which the solutions to the simulations are stable is known as the critical timestep. Different criteria have been defined to calculate the critical timestep, but the Rayleigh critical timestep (*ΔT_Rayleigh_*) is used most frequently. These criteria are calculated as a function of the radius (*R*), the density (*ρ*), the shear modulus (*G*), and the Poisson ratio (*υ*) of the particles:

(1) $$∆T_{Rayleigh}=\frac{\pi R}{0.163\upsilon +0.8766}\sqrt{\frac{\rho }{G}}$$

The displacements of each particle are obtained by explicitly integrating Newton’s differential equations of motion:

(2) $$m_{i}\frac{d^{2}}{dt^{2}}x_{i}=\underset{j}{\sum }\left(F_{n,ij}+F_{t,ij}\right)+m_{i}g$$

(3) $$I_{i}\frac{d\omega_{i}}{dt}=\underset{j}{\sum }\left(T_{ij}\right)$$

where *m_i_*, *I_i_*, *x_i_*, and *ω_i_* are the mass, the moment of inertia, the position, and the angular velocity of particle *i*, respectively; *g* is the acceleration due to gravity, and *F_n,ij_*, *F_t,ij_*, and *T_ij_* are the normal force, the tangential force, and the torque between particles *i* and *j*, respectively. The effect of rolling friction was ignored in this work, so its calculation is not shown in this section.

The force-displacement laws, named contact models, are used to calculate forces between particles (*F_n,ij_* and *F_t,ij_*). In this work, a modification of the Hertz-Mindlin contact model was used. This consists of the addition of a normal cohesion force (*F_n,cohesion,ij_*). Therefore, *F_n,ij_* and *F_t,ij_* were calculated as follows:

(4) $$F_{n,ij}=F_{n,HM,ij}+F_{n,cohesion,ij}$$

(5) $$F_{t,ij}=F_{t,HM,ij}$$

where *F_n,HM,ij_* and *F_t,HM,ij_* are the normal and tangential forces between particles *i* and *j*, respectively, calculated using the Hertz-Mindlin contact model.

In the Hertz-Mindlin model [29], the normal force is based on the Hertzian contact theory, the Mindlin and Deresiewicz theory is used to calculate the tangential force, and the tangential friction force is calculated according to the Coulomb law of friction. In addition to these forces, a damping component is implemented. The following equations include all these components:

(6) $$F_{n,HM,ij}=\frac{4}{3}E_{ij}\delta_{n,ij}^{\frac{3}{2}}\sqrt{R_{ij}}-\sqrt{\frac{20}{3}}\frac{\left(ln\;e\right)}{\sqrt{{ln}^{2}\;e+\pi^{2}}}{\left(m_{ij}E_{ij}\sqrt{R_{ij}\delta_{n,ij}}\right)}^{\frac{1}{2}}v_{n,ij}$$

(7) $$F_{t,HM,ij}=min\left[\mu_{s,ij}F_{n,HM,ij}\left|-8G_{ij}\delta_{t,ij}\sqrt{R_{ij}\delta_{n,ij}}-\sqrt{\frac{80}{3}}\frac{\left(ln\;e\right)}{\sqrt{{ln}^{2}\;e+\pi^{2}}}{\left(m_{ij}G_{ij}\sqrt{R_{ij}\delta_{n,ij}}\right)}^{\frac{1}{2}}v_{t,ij}\right|\right]$$

where *δ_n,ij_* and *δ_t,ij_* are the normal and tangential overlap between particles *i* and *j*, respectively; *v_n,ij_* and *v_t,ij_* are the normal and tangential components of the relative velocity between particles *i* and *j*, respectively; *µ_s,ij_* is the coefficient of static friction between particles *i* and *j*; *E_ij_* is the equivalent Young’s modulus; *G_ij_* is the equivalent shear modulus; *R_ij_* is the equivalent radius; and *m_ij_* is the equivalent mass. They are defined as:

(8) $$\frac{1}{E_{ij}}=\frac{1-\nu_{i}^{2}}{E_{i}}+\frac{1-\nu_{j}^{2}}{E_{j}}$$

(9) $$\frac{1}{G_{ij}}=\frac{2-\nu_{i}}{G_{i}}+\frac{2-\nu_{j}}{G_{j}}$$

(10) $$\frac{1}{R_{ij}}=\frac{1}{R_{i}}+\frac{1}{R_{j}}$$

(11) $$\frac{1}{m_{ij}}=\frac{1}{m_{i}}+\frac{1}{m_{j}}$$

where *E_i_*, *G_i_*, *υ_i_*, *R_i_* and *m_i_* are the Young’s modulus, shear modulus, Poisson’s ratio, radius and mass of the particle *i*, respectively; *E_j_*, *G_j_*, *υ_j_*, *R_j_* and *m_j_* are the Young’s modulus, shear modulus, Poisson’s ratio, radius and mass of the particle *j*, respectively.

The normal cohesion force is calculated as follows:

(12) $$F_{n,cohesion,ij}=k_{ij}A_{ij}$$

where *k_ij_* is the cohesion energy density and *A_ij_* is the contact area between particles *i* and *j*. The contact area is calculated though Equations (13) or (14) depending on the contact model.

### 2.2. DEM Simulations

The DEM simulations were performed using two different software programs: EDEM^®^ 2.7.2 (DEM Solutions Ltd., Edinburgh, UK) and LIGGGHTS^®^ version LIGGGHTS-PUBLIC 3.3.1 (DCS Computing GmbH, Linz, Austria). The postprocessing of the simulations that were run in LIGGGHTS was performed using PARAVIEW version 4.4.0 64-bit (Kitware Inc., New York, NY, USA). The simulations were conducted on a server with 64 GB of RAM with a 2 × Intel Xeon Quad-core 2.0 GHz E5504 CPU (© Intel Corporation, Santa Clara, CA, USA). The simulations in LIGGGHTS were run on a single processor. In contrast, the simulations in EDEM were run in batch mode using four cores.

To search for equivalent cohesion contact models in both DEM packages, three contact models were used in this work. The linear cohesion contact model was used in the simulations run in EDEM. In LIGGGHTS, the simplified Johnson-Kendall-Roberts (SJKR) and modified SJKR (SJKR2) models were applied. The reason for choosing these contact models was that they all calculate the cohesion force as a normal force that is proportional to the contact area, as shown in Equation (12). Other cohesion contact models are available in both software programs, but they consider other forces, such as the liquid bridge force (e.g., the Hertz-Mindlin with JKR cohesion model in EDEM or the easo/capillary/viscous and washino/capillary/viscous models in LIGGGHTS). This means that other parameters, different from the cohesion energy density and contact area, are considered for the calculation of the force. Therefore, the equations that define the cohesion force are also different. Although the three contact models that were used in is work seem to be equivalent, they calculate the contact area using different methods. The contact area in the SJKR2 and linear cohesion models is calculated as a simplification of the contact area used in the SJKR model. The latter uses the area of the circle corresponding to the intersection of two spheres with different diameters [30], whereas the first considers the equivalent radius. The contact area (*A_ij_*) between particles *i* and *j* is calculated in each contact model as follows:

(13) $$A_{ij\left(SJKR\right)}=\frac{\pi }{4}\frac{\left(-d+R_{i}+R_{j}\right)\left(d+R_{i}-R_{j}\right)\left(d-R_{i}+R_{j}\right)\left(d+R_{i}+R_{j}\right)}{d^{2}}$$

(14) $$A_{ij\left(SJKR2,linear\;cohesion\right)}=2\pi \delta_{n,ij}R_{ij}$$

where *R_i_* and *R_j_* are the radii of particles *i* and *j*, respectively; *d* is the distance between their centers; *δ_n,ij_* is the normal overlap between particles *i* and *j* that is calculated as *R_i_ + R_j_ − d*; and *R_ij_* is the equivalent radius, see Equation (10). Despite this difference, it could be interesting to analyse how the results and computational costs are influenced by this factor.

**Figure 1:**
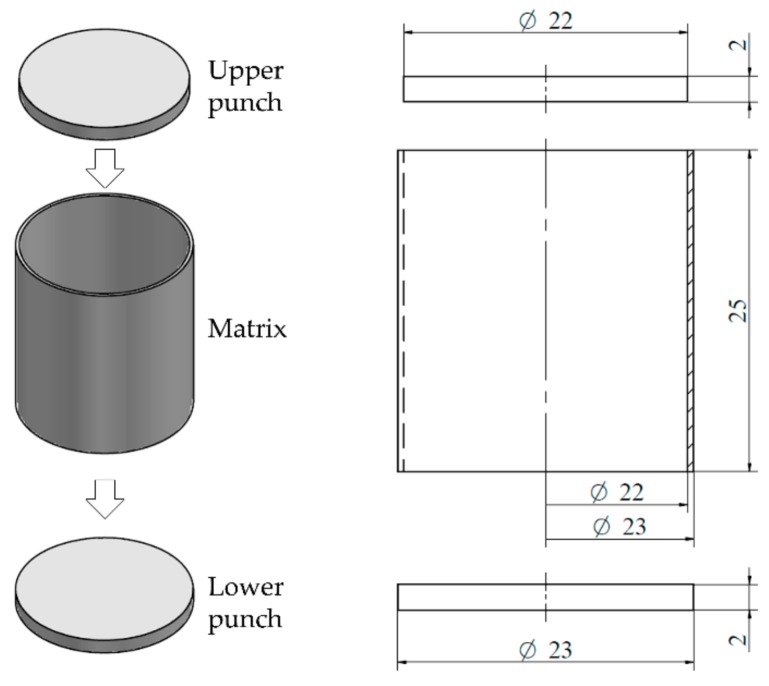
Geometries used in discrete element method (DEM) simulations. Dimensions are expressed in mm.

The compaction mechanism in the uniaxial presses used to form compacts is generally composed of two punches and a matrix. The punches compact the powder into the required shape that is determined by the shape of the matrix when pressure is applied. The geometries of the components that were used in this work and their dimensions are depicted in Figure 1.

**Figure 2:**
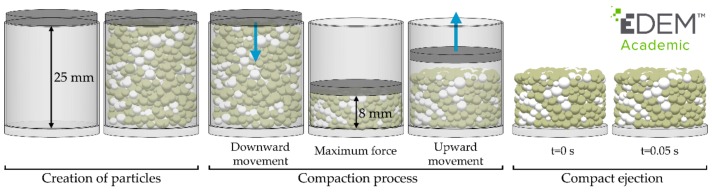
Stages of the simulated process.

The process that was simulated in this work consisted of the three following stages, as shown in Figure 2: the creation of the particles, the compaction process, and compact ejection. During the creation process, the particles were created in a cylindrical factory. This factory was a virtual geometry located in the initial assembly of the physical components (punches and matrix). At this moment, the upper punch was located 25 mm away from the lower punch. After the particle creation was completed, the compaction process began by moving down the upper punch with a constant velocity. This movement stopped when the distance between the punches was 8 mm. At this moment, the upper punch applied the maximum force. Then, the upper punch began its upward movement with the same velocity. The movement ceased when the upper punch was placed at a height of 16 mm. Finally, the matrix was removed, and the simulation process ended after waiting for 0.05 s. Although in real applications the punch remains at its lower position during a dwell time, this aspect was not simulated in order to reduce the computational costs. By using an appropriate dwell time, some of the defects in the compacts are reduced [31,32]. However, the quality of the compacts was not analysed in this work. Another difference between the simulations performed in this work and real applications is that the compact is ejected from the matrix in real processes, whereas the matrix disappeared in the simulations. Consequently, the effect of the friction between the compact and the wall of the matrix was ignored in the simulations. Nevertheless, the processes that were simulated in this work were valid for comparing the contact models and analysing their applicability to compact granular materials. To simulate this process, the geometries of each component (punches and matrix) were generated on computer-aided design (CAD) software (Solid Edge Version 18, © UGS Corp., Plano, TX, USA) and then imported into the DEM software as standard triangle language (STL) files.

Two granular materials were simulated in this work. Their properties are shown in Table 1. Although the particle sizes of these materials and some of their properties were unreal, they mimicked the simulation of two real materials. The Young’s modulus of the real materials are approximately 200–250 GPa, but lower values are usually used to simulate materials at a reasonable computational cost [33]. The reduction in the computational cost was crucial in this work because of the number of simulations that were performed. Preliminary simulations were performed to determinate some of the interaction parameters. The effect of the rolling friction on the results was analysed by comparing different simulations, in which values in the range of 0 to 0.05 were assigned to the materials. The results of these simulations revealed a small influence of the rolling friction. Therefore, the effect of the rolling friction was ignored in all cases. The coefficient of static friction was 0.5, and the coefficient of restitution was 0.2 for all possible interactions (particle–particle, particle–wall, and wall–wall). The cohesion effect was only applied to the interparticle contacts. The value of cohesion energy density was 3 × 10^6^ J/m^3^ in all of the simulations. The cohesion energy density is strongly dependent on some of the properties of the materials, such as the Young’s modulus and particle size. Its calibration is necessary to accurately simulate the cohesive behavior of real materials. However, the purpose of this work was to analyse the capability of different cohesion contact models to simulate the compaction process. Therefore, the calibration of the cohesion energy density in this work involved determining the optimal value that allowed the formation of a compact. The value used in this work was determined after performing several simulations with values of cohesion energy densities in the range of 1 to 5 × 10^6^ J/m^3^. These simulations showed stability problems at high values, whereas low values were not capable of keeping the particles joined after the compaction.

**Figure 3:**
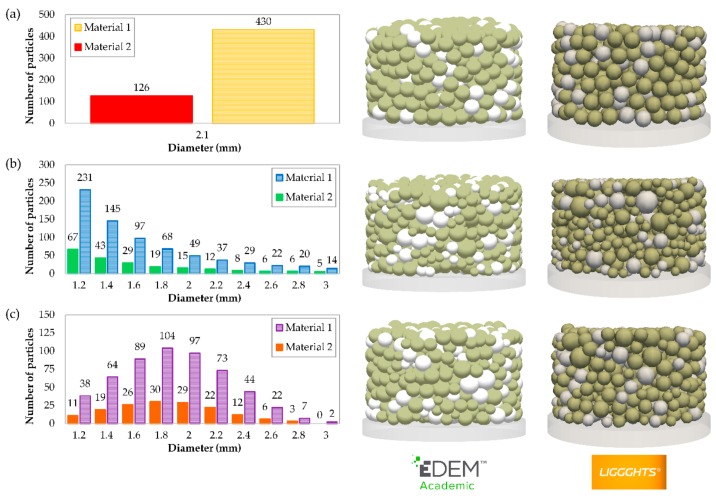
Number of particles used in the DEM simulations and the appearance of compacts obtained in both software programs with different particle size distributions (PSDs): (**a**) monosized particles (MP), (**b**) uniform distribution (UD), and (**c**) normal distribution (ND).

A combination of different materials is commonly used in the metallurgical industry [34]. Thus, a mixture of Materials 1 and 2 in weighted proportions of 80% and 20%, respectively, was used. Material 1 was colored beige and Material 2 was colored white, as shown in Figure 2 and Figure 3, to distinguish between the particles of each material. To examine the influence of the size of particles, three different PSDs were analysed. These PSDs were as follows: monosized particles (MP), uniform distribution (UD), and normal distribution (ND). The sizes of all the particles were the same in the first PSD (MP). The mixture of the second one (UD) consisted of the same particle size proportions by weight. The sizes of the particles followed a normal distribution in the last one (ND). Figure 3 shows the number of particles of each size that were created to simulate the mixtures using the different PSDs.

The simulations were initially run in LIGGGHTS. After visualization of the simulated system in PARAVIEW, the simulations were conducted in EDEM. Thus, the simulations in EDEM were modelled exactly like their homologues in LIGGGHTS. This was possible due to controlling all the parameters and dynamics of the models throughout the EDEM interface. For example, both software programs enabled the automatic creation of a set of particles with different PSDs (uniform distribution, Gaussian distribution, etc.). However, the number of particles of each size could be varied in the different simulations using these options. Thus, none of the automatic options were used in EDEM to create the particles; instead, the number of particles of each size was introduced manually. As a result, the same number of particles of each size was used in both programs. However, the positions of the particles were not identical in all the simulations with the same PSD because the particles were created randomly in both DEM simulators. This difference had a negligible impact on the results.

### 2.3. Methodology

This work analysed the relationship between five parameters introduced in the simulations (timestep, conversion tolerance, speed of the upper punch, PSD, and contact model) and three critical factors that determined the goodness of the simulations and their efficiency: the maximum force applied by the upper punch, the percentage of eliminated contacts (related to the final appearance of the compact), and the computational cost.

First, the influence of the timestep was examined. For this purpose, six simulations (Table 2, setups no. 1–6) using different timesteps were conducted for all PSDs and contact models. The timestep values were approximately 5%, 10%, 15%, 20%, 25%, and 30% of the Rayleigh timestep. However, the Rayleigh timestep was higher for the MP distribution than for UD and ND because the latter used smaller particles. Moreover, it was necessary for the timestep values to be divisors of the values of the target save interval (0.001 s in these simulations) so that the results were obtained at exactly the same time. For these reasons, the values of the timesteps in the simulations of the MP distribution were different from those used in the UD or ND. In addition, the computational costs required to complete these simulations were analysed to obtain the optimal value for the Rayleigh timestep percentage. 

**Figure 4:**
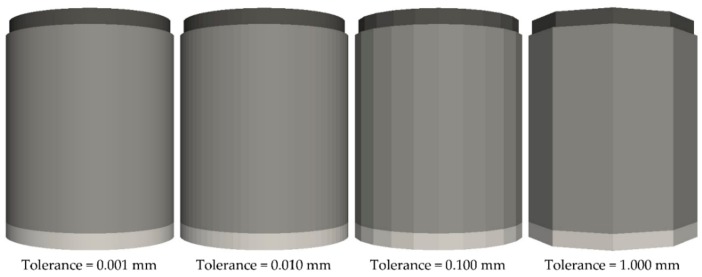
Geometries used to analyse the effects of the conversion tolerance.

The use of different geometries was analysed. For this purpose, the geometries corresponding to the punches and matrix were generated on CAD software and exported as STL files using different conversion tolerances (Table 2, setups no. 2 and 7–9). The mesh sizes of the geometries increased as the tolerance increased. Therefore, the geometries with smaller meshes were more similar to the theoretical cylindrical geometries, but they became prisms with a lower number of lateral faces when the sizes of the meshes were greater, as shown in Figure 4. In this work, the effects of using four conversion tolerances on the results and computational cost were analysed to determine the optimum value. Finally, several simulations (Table 2, setups no. 2 and 10–17) were conducted to examine the effect of the punch speed. The simulation time of the simulations of the compaction process increased proportionally as the speed of the punch decreased (Table 2). Therefore, the computational cost also increased. For this reason, we considered how this parameter influenced the results of the simulations and their computational costs to determine the maximum speed that could be applied to obtain adequate results by employing an acceptable time.

The influence of the PSD was analysed by comparing the results of all simulations. The similarities and differences between the results obtained with the different contact models were examined in all of the simulations.

The three factors mentioned above (maximum force, percentage of eliminated contacts, and computational cost) were compared in all cases. The maximum force applied by the upper punch during the compaction process was analysed. The maximum force value was attained when the upper punch was located 8 mm from the lower punch. This factor can be considered the most important, as some of the main defects in the compacts are generated by the application of excessive or insufficient force. Consequently, the force is one of the parameters that is usually controlled in experimental testing. In addition, the appearance of the compacts after they were compacted was examined. To quantify this quality of the compacts, the percentage of eliminated contacts from 0.01 s to 0.05 s after the matrix was removed was calculated. This parameter is related to the final appearance of the compacts because a greater percentage of eliminated contacts indicates that the particles have been separated and the forces that kept them joined have disappeared. If this occurs, the particles could fall and form a heap of particles instead of a compact. Finally, the computational costs of the simulations were compared. Thus, a combined examination of these three factors was performed to determine the optimal values of the timestep, conversion tolerance, and punch speed. The optimal values of these parameters were those that provided adequate results (maximum force and percentage of eliminated contacts) using the lowest computational cost.

## 3. Results and Discussion

### 3.1. Effect of the Timestep

**Figure 5:**
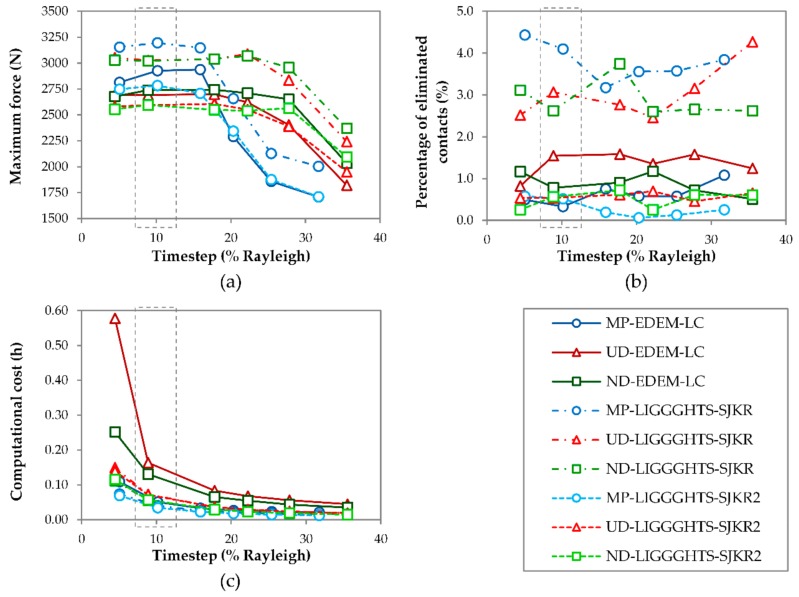
Effect of the timestep on (**a**) the maximum force applied by the punch, (**b**) the percentage of eliminated contacts after the matrix has been removed, and (**c**) the total computational cost.

Figure 5 shows the effects of the timestep on the maximum force applied by the punch, the percentage of eliminated contacts after the matrix was removed, and the computational cost of the simulations.

As evident in Figure 5a, the maximum force was dependent on the timestep that was fixed in the simulations. The trend shown in all of the curves is the same. The maximum force remained nearly constant as the timestep increased at low values. However, the force decreased as the timestep increased at higher values. More specifically, these values were approximately equal to 15% of the Rayleigh timestep in the cases of MP and UD, but in the case of ND, the change in the curve was produced near the 25% Rayleigh timestep. This behavior was more evident in the case of MP. The maximum forces that were applied by the punch in the simulations that used the SJKR model were greater than their homologues using the SJKR2 and linear cohesion models, as Figure 5a illustrates. By comparing the calculations of the contact area in each model, the value of the contact area that was calculated using Equation (13) (SJKR model) was slightly lower than those calculated using Equation (14) (SJKR2 and linear cohesion models) for the same pair of particles. Consequently, the cohesion force acting on a pair of particles using the SJKR model was lower than that obtained using the SJKR2 or the linear cohesion model. This lowers the updated normal overlap between the pair of particles in the SJKR model than in the other models if only the cohesion force was acting on the particles. Therefore, the average overlap in an assembly of particles was also lower for the SJKR model when external forces were not acting upon it. As a consequence, the volume that the assembly of particles occupies was greater for that model. The volume occupied by the particles when the punch applied the maximum force was the same for all contact models, i.e., the overlap was necessarily the same for all cases. For that reason, the punch must apply a greater force when the SJKR model is used to achieve the same average overlap obtained by the SJKR2 or the linear cohesion model. Despite this difference, the curves obtained from the homologue simulations were similar, regardless of the contact model used. The behavior of all of these curves indicates that the simulation models converge at low timesteps, regardless of the PSD and contact model used. This may be due to an error in the calculation of the position of the punch or its force when a large timestep is fixed.

Figure 5b shows the percentage of eliminated contacts during the compact ejection. This parameter quantifies the stability of the particles, which is related to the compact’s final appearance. Two groups were clearly identified. The results obtained with EDEM and using the SJKR2 model in LIGGGHTS indicated a greater compact stability in comparison with the results obtained using the SJKR model. The percentage of eliminated contacts was greater in the latter. This was due to the difference between the calculations of the cohesion force, as mentioned above. A small expansion of the compact occurred after the matrix was removed in all cases as a consequence of the cessation of the confinement force. Therefore, the distance between particles increased due to this expansion, and some of the particles that were in contact during the confinement stopped being in contact afterward. The expansion ended when the tensional state stabilized. At this moment, the contact force between the particles was lower when the SJKR model was used because the cohesion contact force was also lower. This reduced the overlap between the particles, i.e., the distance between the particles was greater. For that reason, the number of eliminated contacts was higher when the SJKR model was applied. No relationship was found between the PSD or timestep used and the percentage of eliminated contacts.

Regarding the effect of the timestep on the computational cost, cost decreased as the timestep increased, as shown in Figure 5c. This occurred because the number of iterations that were necessary for completing a determined simulation was lower when a large timestep was used than when a low value was adopted. The slopes of all the curves decreased heavily at 5–15% Rayleigh timesteps and decreased slightly from 15% onward, especially in simulations using EDEM. The computational costs required by the simulations that were run in EDEM were greater than those required for their homologue simulations using the two contact models in LIGGGHTS, despite the fact that the first simulations ran under four cores and the latter only used one core. The difference between the computational costs for both types of software using the same number of cores were assumed to be greater. However, the use of different numbers of cores did not have a considerable impact on the computational costs for the same simulation in EDEM. This could be because the files that save the results in EDEM increase at each data save interval; that is, it is necessary to read and write those files as many times as there are data points. However, in LIGGGHTS, a new result file is created at each data save interval. The computational costs required for both packages would be reduced if graphics processing unit (GPU) was used.

In view of these results, 10% Rayleigh timestep was determined to be the optimum value because the maximum force remained constant until a 15% Rayleigh timestep in all cases. The percentage of eliminated contacts was not related to the timestep and the major diminution of the computational cost occurred between the 5% and 10% Rayleigh timesteps. The selection of this timestep matches the range of critical timesteps determined by Thakur et al. [35]. Although the optimal Rayleigh timestep value of 10% was used for the set of simulations shown in Table 2 in setups no. 1–6, the value might be different for the simulation of other materials or operating conditions (punch speed).

### 3.2. Effect of Conversion Tolerance

**Figure 6:**
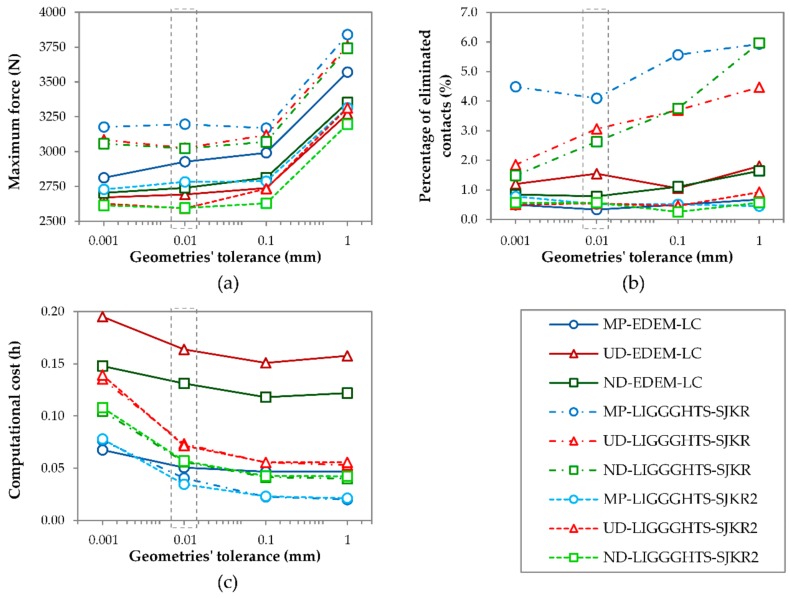
Effect of the conversion tolerance on (**a**) the maximum force applied by the punch, (**b**) the percentage of eliminated contacts after the removal of the matrix, and (**c**) the total computational cost.

Figure 6 shows the effect of using different conversion tolerances to export the three-dimensional (3D) models to STL files on the maximum force applied by the punch, the percentage of eliminated contacts after the removal of the matrix, and the computational cost.

The maximum force increased with the conversion tolerance, regardless of the PSD simulated or the contact model used, as illustrated in Figure 6a. This trend arose because the cross-section of the geometries decreased as the tolerance increased, as shown in Figure 4. The maximum force varied slightly between tolerance values of 0.001 and 0.1 mm, but increased considerably from 0.1 mm to 1 mm. This occurred because the prism corresponded to the geometry, where 1 mm of tolerance only had 10 faces. The maximum force values of the simulations that used the linear cohesion and SJKR2 models were lower than those obtained using the SJKR model, as shown in Figure 5a.

Figure 6b plots the percentage of eliminated contacts during the compact ejection. The results of the simulations run in EDEM and LIGGGHTS using the SJKR2 model were almost unaffected by the conversion tolerance. However, the percentage of eliminated contacts increased considerably with the tolerance when the SJKR model was used, except for the MP distribution. This probably occurred because the overlaps between particles were greater at the beginning of the ejection process (before the matrix was removed) as the conversion tolerance was greater because the cross-section was smaller, and the particles had smaller volumes. This was the case for all the contact models, but the effect was more marked in the simulations that used the SJKR model because the cohesion force in this model was lower than that calculated for the linear cohesion and SJKR2 models. Thus, the confinement force was necessarily greater for the SJKR model. When this force ceased, the expansion of the disc simulated under the SJKR model was greater than in the other simulations. The percentage of eliminated contacts using the SJKR model was generally greater for the MP distribution than for the other models and was less affected by the conversion tolerance. No relationship was found between the PSD and percentage of eliminated contacts.

The difference in the number of faces affected the computational cost, as illustrated in Figure 6c. The time simulation decreased as the conversion tolerance increased. This occurred because the number of elements that compounded the meshes increased as the mesh size decreased; that is, the number of elements increased as the tolerance decreased. Therefore, the simulation times were longer when the tolerance was lower because the DEM simulators detected the contact between the particles and surfaces (mesh elements). When the mesh sizes of the geometries were extremely small, the DEM simulators had to check more elements to identify whether the particles were in contact with them. This was the case for the geometry with 0.001 mm tolerance, where the simulation time was considerably higher than the time required to simulate the geometry with 0.01 mm tolerance. The effect of the conversion tolerance on the computational cost was lower in the simulations using EDEM than in their homologues in LIGGGHTS, regardless of the PSD simulated.

The maximum force increased when the tolerance decreased, the percentage of eliminated contacts remained constant (except for the SJKR model) and the computational cost decreased with the tolerance, especially from 0.001 mm to 0.01 mm. For these reasons, when considering the effect of the conversion tolerance on the maximum force and computational cost, a tolerance of 0.01 mm was considered optimal.

### 3.3. Effect of Punch Speed

**Figure 7:**
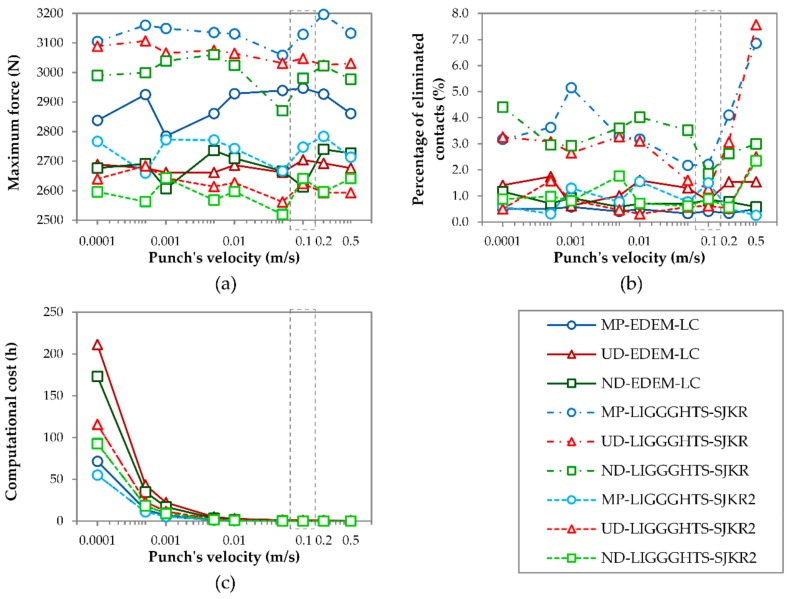
Effect of the speed of the punch on (**a**) the maximum force applied by the punch, (**b**) the percentage of eliminated contacts after removal of the matrix, and (**c**) the total computational cost.

Figure 7 shows the effect of moving the punch at different velocities on the maximum force it applies, the percentage of eliminated contacts after removal of the matrix, and the computational cost.

As shown in Figure 7a, the maximum force values fluctuated with the velocity of the punch; thus, no relationship exists between the speed of the punch and the force it applies in the range of velocities that were considered in this work. However, the results obtained in LIGGGHTS using the SJKR model showed higher values than their homologues using the SJKR2 model or the linear cohesion model from EDEM. This is in accordance with the results shown in Figure 5a and Figure 6a. The difference between the results obtained using the linear cohesion and SJKR2 models was greater in the case of MP than in the other PSDs.

As Figure 7b displays, for the SJKR model, the percentage of eliminated contacts after the matrix was removed fluctuated with a punch speed up to 0.01 m/s. Then, the percentage decreased from 0.01 to 0.1 m/s. After that, it increased and attained its maximum value at the fastest speed (0.5 m/s) in several cases. The minimum attained at 0.1 m/s means that the compacts obtained at that punch speed were more stable than those compacted at other speeds for this contact model. However, this minimum was not found for the other contact models, where the percentage of eliminated contacts fluctuated from 0.0001 m/s to 0.5 m/s. The amplitude of the fluctuations was lower in these latter cases. In addition, the percentage of eliminated contacts was lower in the simulations that used the linear cohesion and the SJKR2 models than in the simulations corresponding to the SJKR model, including the simulations with a punch speed of 0.1 m/s. Although a velocity of 0.2 m/s was used in the previous simulations (Table 2, setups no. 1–9), the results shown in Figure 5b and Figure 6b are considered valid because the SJKR model showed a higher percentage of eliminated contacts than the other models for all of the punch speeds. The same can be observed in Figure 5b and Figure 6b. The other reason is that it is not necessary to use the optimal values to compare the different contact models; the conditions are only required to be equal.

The relationship between the computational cost and punch speed was clear: the computational cost increased exponentially as the speed of the punch decreased (Figure 7c), regardless of whether the PSD or the contact model was used. This was because the simulation time increased from 0.05 s (when the punch moved at 0.5 m/s) to 250 s (when the punch’s speed was 0.0001 m/s), as shown in Table 2. Therefore, this latter simulation required 5000 times as many interactions as required by the first one.

In brief, no relationship was observed between the maximum speed of the punch and the maximum force that it applies. The percentage of eliminated contacts was lowest 0.1 m/s, and after that, the percentage increased for the SJKR model. No relationship was found for the other contact models. The computational cost decreased exponentially as the speed of the punch increased. As a consequence, a punch speed of 0.1 m/s was the optimal value for all of the contact models.

### 3.4. Effect of the PSD

In general, PSDs can be sorted into MP, UD, and ND according to their maximum force values in descending order, as illustrated in Figure 5a, Figure 6a, and Figure 7a, respectively. The rearrangement of the particles during the confined compression is affected by the PSD, as indicated by Wiacek and Molenda [19]. This reorganization of particles is easier in UD and ND than in MP because of the presence of large voids filled by the smaller particles. This increases the volume that the particles occupy in MP compared to the volume occupied by particles in UD and ND after rearrangement. Therefore, it was necessary to apply a greater force to the MP material to compress it into a cylindrical cavity with a diameter of 22 mm and a height of 8 mm. This behavior means that the powder related to the MP distribution is less compressible than the UD and ND powders. This is in accordance with the reduction in porosities of each mixture after their compaction. In the current study, the mean initial porosities were 54.5%, 52.5%, and 53.8% in the compacts with MP, UD, and ND mixtures, respectively, for setup no. 16 (Table 2). Their mean final porosities were 44.7%, 42.0%, and 43.5%, respectively. Therefore, reductions of 9.9%, 10.5%, and 10.3% were obtained for the MP, UD, and ND powders, respectively. Although the differences in the porosity reductions for the different mixtures were small, these results confirm that the MP powder is less compressible than the others. In contrast, it was not clear if the percentage of eliminated contacts was related to the PSD. Regarding the computational cost, the PSDs can be sorted in ascending order as follows: MP, ND, and UD. This classification is due to the number of particles that were simulated in each case and the minimum particle size they contain. First, the lowest computational costs were required for the MP distribution. This PSD contained 556 particles with a diameter of 2.1 mm. Second, the ND was composed of 698 particles with diameters between 1.2 and 3 mm and demanded intermediate computation time. Finally, although the UD used the same size range of particles as ND, the UD required the most computational time because it used a greater number of particles (922).

### 3.5. Comparison between the Contact Models

**Figure 8:**
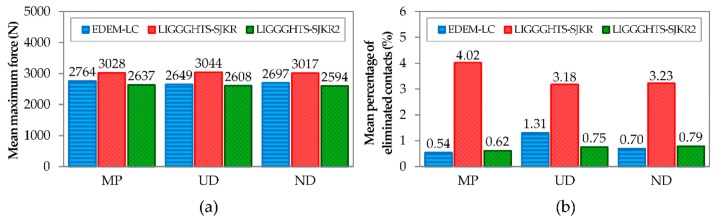
Mean values of (**a**) the maximum force and (**b**) the percentage of eliminated contacts.

Figure 8 shows the mean results obtained using each model for different PSDs. As shown in Figure 8a, the mean values of maximum force were similar for all contact models. However, the results that were obtained from LIGGGHTS using the SJKR2 model and from EDEM (linear cohesion) were more similar than those obtained using the SJKR model. The maximum difference between the first models (SJKR2 and linear cohesion) was lower than 150 N. The minimum difference between the results corresponding to the SJKR model and the results of the other models was greater than 250 N. Regarding the percentage of eliminated contacts, a greater difference was found between the results obtained from the SJKR model and those obtained from the linear cohesion and SJKR2 models, as shown in Figure 8b. The mean percentage of eliminated contacts was higher than 3% when the SJKR model was used, but lower than 1.5% for the other contact models. This means that the compacts were more stable in the simulations that used the linear cohesion and SJKR2 models. In view of these results, the linear cohesion and SJKR2 models can be considered equivalent contact models.

## 4. Conclusions

Several simulations of a compaction process were performed to examine the capability of certain contact models. For this purpose, three contact models were tested. All of them allowed the formation of a compact via particle cohesion. Some differences were observed between them. This enabled us to obtain equivalent DEM models with two different simulators: EDEM and LIGGGHTS. In addition, different timestep, conversion tolerance, and punch speed values were used. Three PSDs were simulated to investigate the influences of these parameters on the results obtained using each PSD. Thus, we established the following conclusions.

The selection of the timestep determines the maximum force that the punch applies due to the inaccuracy of the calculation when a long timestep is chosen. The results showed that the maximum force is lower when the timestep is higher. The computational cost decreases with the increase in this simulation parameter. However, the percentage of eliminated contacts does not depend on the selected timestep. A 10% of Rayleigh time was adequate to simulate the compaction process using the materials and the contact models employed in this work.

Concerning the conversion tolerance, the force applied by the punch increases as the tolerance of the geometries increases because the cross-section is smaller when the tolerance is higher. The behavior of the percentage of eliminated contacts differs depending on the applied contact model. The conversion tolerance does not influence the percentage of eliminated contacts when the linear cohesion or SJKR2 model is used. However, the percentage of eliminated contacts increases as the tolerance rises when the SJKR model is selected. Contrary to the maximum force, the computation cost decreases as the conversion tolerance increases because the number of faces is lower in those geometries where the tolerance is higher. For these reasons, the tolerance that is recommended for converting the 3D models to STL files for the geometries used in this work is 0.01 mm.

The maximum force fluctuates with the punch speed. Therefore, no relationship between the two was observed. The percentage of eliminated contacts fluctuated for the linear cohesion and SJKR2 models, but a minimum was attained at a speed of 0.1 m/s for the SJKR model. The computational cost is strongly influenced by the punch speed. Consequently, the maximum speed for moving the punch is 0.1 m/s for the contact models used in this work.

Concerning the PSD of the materials, the maximum forces are obtained for the MP distribution because the particle rearrangement is more challenging in this context than it is in other PSDs because the large voids between particles were filled by the smaller particles in UD and ND distributions. However, there is no relationship between the percentage of eliminated contacts and the PSD. The MP distribution has the lowest computational costs because a small number of large particles is created. However, a larger number of small particles is found in the other PSDs. The simulations that use the UD distribution have the highest computational costs because they use the largest number of particles.

Finally, the conclusions relating to the contact models and software packages are as follows. Although the results obtained using the three contact models were similar, a greater difference was found in the results related to the SJKR model. Therefore, the linear cohesion model implemented in EDEM and the SJKR2 model in LIGGGHTS can be considered equivalent.

This work focused on the differences and similarities between several contact models, but this study was intended to be a starting point for the study of the compaction process. This work does not propose models that simulate real systems, but aimed to provide a better understanding of the dependence between the parameters used and the results obtained. The optimal values obtained in this work might be different for other applications, but the practitioners might use them initially to study their particular problems. Consequently, the application of the equivalent contact models for simulating the compaction of real materials requires additional investigation, such as the calibration of the simulation models by comparing them with experiments. Once the models have been validated, they might be useful for understanding the compaction process and finding solutions to the problems that usually occur during the formation of compacts.

## Figures and Tables

**Table 1:** Summary of material properties used in the DEM simulations.

Material Property	Material 1	Material 2	Wall
Mean diameter (mm)	2.1	2.1	-
Density (kg/m^3^)	3500	3000	8000
Young’s modulus (Pa)	2.5 × 10^8^	2.5 × 10^8^	2.0 × 10^8^
Poisson’s ratio	0.25	0.25	0.25

**Table 2:** Setup of DEM simulations.

Setup Number	Timestep (s)		Timestep (% Rayleigh)	Conversion Tolerance (mm)	Speed of Punch (m/s)	Total Simulation Time (Compaction + Ejection) (s)	
	MP	UD/ND					
1	1.0 × 10^−6^	5.0 × 10^−7^	~5	0.01	0.2	0.175	(0.125 + 0.05)
2	2.0 × 10^−6^	1.0 × 10^−6^	~10	0.01	0.2	0.175	(0.125 + 0.05)
3	3.125 × 10^−6^	2.0 × 10^−6^	~15	0.01	0.2	0.175	(0.125 + 0.05)
4	4.0 × 10^−6^	2.5 × 10^−6^	~20	0.01	0.2	0.175	(0.125 + 0.05)
5	5.0 × 10^−6^	3.125 × 10^−6^	~25	0.01	0.2	0.175	(0.125 + 0.05)
6	6.25 × 10^−6^	4.0 × 10^−6^	~30	0.01	0.2	0.175	(0.125 + 0.05)
7	2.0 × 10^−6^	1.0 × 10^−6^	~10	0.001	0.2	0.175	(0.125 + 0.05)
8	2.0 × 10^−6^	1.0 × 10^−6^	~10	0.1	0.2	0.175	(0.125 + 0.05)
9	2.0 × 10^−6^	1.0 × 10^−6^	~10	1.0	0.2	0.175	(0.125 + 0.05)
10	2.0 × 10^−6^	1.0 × 10^−6^	~10	0.01	0.0001	250.05	(50 + 0.05)
11	2.0 × 10^−6^	1.0 × 10^−6^	~10	0.01	0.0005	50.05	(50 + 0.05)
12	2.0 × 10^−6^	1.0 × 10^−6^	~10	0.01	0.001	25.05	(25 + 0.05)
13	2.0 × 10^−6^	1.0 × 10^−6^	~10	0.01	0.005	5.05	(5 + 0.05)
14	2.0 × 10^−6^	1.0 × 10^−6^	~10	0.01	0.01	2.55	(2.5 + 0.05)
15	2.0 × 10^−6^	1.0 × 10^−6^	~10	0.01	0.05	0.55	(0.5 + 0.05)
16	2.0 × 10^−6^	1.0 × 10^−6^	~10	0.01	0.1	0.3	(0.25 + 0.05)
17	2.0 × 10^−6^	1.0 × 10^−6^	~10	0.01	0.5	0.1	(0.05 + 0.05)
